# Extracts from Essays on Chinese Dentistry

**Published:** 1895-05

**Authors:** C. Robbing


ARTICLE VII.

### EXTRACTS FROM ESSAYS ON CHINESE DENTISTRY.

BY A. M. H. (COMMUNICATED THROUGH C. ROBBING, L. D. S., ENG.)

**Written by young Chinese students who are learning English at the Anglo-Chinese College, Foochow, China.**

In China dentistry is not widely practiced, as the dentists not only are unable to supply the lost teeth but also they are not able to arrest the decay of the teeth, therefore I have only concentrated two themes in my mind upon which I will write, viz., the extraction of the teeth and the pretense of catching the tooth worms. Once upon a time on my way to school, while passing the Ming bridge, I chanced to see a personage submitting to an operation of a native dentist, as he had undergone pain in one of his molars for some time. My attention being attracted I took my stand by them to watch narrowly what was going on. The dentist dipped some soft pink substance on his instrument, which was somewhat like a knitting pin, but instead of having pointed ends had blunted ones, and he applied it to the base of his patient's diseased molar. During the meanwhile he put an end to his talk by saying: "Ha! beware! keep your tongue still, lest it will touch the medicine and be injured." In another moment he extracted the diseased molar by means of his forceps, with perfect ease. His patient then gargled his mouth with water in order to prevent the gushing of the blood, and the operation was completed. After all was over, I took my departure and resumed my journey to school with great satisfaction. On inquiry, I found out that the drug which the dentist employed is generally and properly known by the name "Lu sauk-dang" (arsenic,) "the drug of severing bones." Some is white. According to my opinion the difference of its colors is due to the variety of the coloring matter stirred and mixed in the mixture.

The prescription of the medicine necessary in making it and the method of mixing it are hidden in the pith of the minds of the selfish dentists, each of whom has the lot, marked out by his birth, of being secretly communicated by their forefathers with them.

On this account everyone of the personages of whom I have diligently enquired as to how it is composed, and how it is made, alleges that he has not the least idea. Now, I want to state another case; I recollect when I was a stripling being a spectator of a ridiculous dental operation performed by a menial surgeon. His life was that of an itinerant. Generally he made his choice of the open space in front of some club elaborately sculptured, or in the vicinity of some highway, to set out varieties of drugs and ointments, highly burnished knives and crockery, such as canisters, mugs, etc., together with lamps all mingled higgledy-piggledy, pretty much as I have enumerated them. No sooner he struck his gong to attract a crowd than people came flocking to him, and children, as a matter of course, would elbow their way through the throng towards him. After all he offered to extract a tooth free of charge for anyone who would submit to his first operation. A boy of a dozen years old, who was neither well bred nor well-to-do, took advantage of his offer, and stepped forward to have his shaken tooth extracted. Then the surgeon dipped a piece of paper in a mug of Lu-sank-dang (arsenic) and brought it in contact with the tooth. At the same time he enjoined upon him to close his mouth as tightly as possible, and pasted half a dozen strips of paper at one end on his face, leaving the other ends downward. The boy, thus adorned, was such an apparition as is seldom to be met with in broad daylight. After everything was ready, the surgeon befooled the boy, saying, "You should jump as often as I beat the gong, so that your tooth will be fully influenced to cause to drop down by the drug without slight pain." We undoubtedly know that a boy of a dozen years is generally an odd mixture of ignorance to credulity. On account of this he readily followed his advice and jumped as speedily as he beat his gong. The wide throng of spectators of course burst into a laugh, and meanwhile the tooth fell off.

Now, let me advert to the pretence of arresting the tooth worms. One of my relatives was once attacked by a serious cold, and after the cold was broken up by restoring activity to his skin he had a neuralgia having its origin in one of his molars, which gave him such an intense suffering that he could neither eat nor repose, but moaned with a voice so audible and so plaintive that it sent a thrill to the heart of everyone in the house. At first he endeavored to alleviate his pain by holding the solution of wonghu in his mouth, but without avail. On the second day his suffering increased to a remarkable degree, indeed it is impossible even at this distant period to reflect without horror on the miseries of his toothache state. Then a surgeon stepped in and declared that as the molar was affected by the cold which attacked before it was not tractive and that if extracted there would be no end to the bleeding, so that he went his way.

Finally he submitted to an operation of a woman dentist whose agency was to arrest tooth worms. Her general operation is as follows: A chopstick and a silver pin are the only instruments she requires in her normal act. She is willing to exhibit them to any one who conceives an inclination of discerning her trickery. She brings the chopstick in contact with the diseased tooth, and cautiously pokes it through with the pin in search of the odious worms; after awhile scrapes out a lump of yellow minute worms on the chopstick and immerses it in a cup of water. Each lump consists of from ten to fifteen worms, and sometimes two to three hundred worms are scraped in an immense number of lumps, *if the patient makes an exact bargain at first that the fee should be defrayed according to number of worms scraped.* She declares that each lump of worms abide in the same domicile located in the diseased tooth.

The general fee is four hundred cash (about 1s. 2d.) and only the poor may take advantage of being in penury to pay two hundred cash. With reference to my relative, the treatment relieved pain for a couple of days. After the elapse of that time he was in an intolerable agony again. No relief could be secured save by a fresh resort to that lady's booth and another submission to her operation. His toothache was treated in this way time and again, but was not eradicated. Ultimately the neuralgia of the tissues of his diseased molar was broken by following the medical advice of one of his acquaintances, that diets which were cold in nature were the best remedies of the disease of the kind.

Why is it her treatment can relieve the toothache for sometimes, or even eradicate entirely? It is because, I suppose, that the pricks of the pin have the effect of bringing the poisonous blood out of the diseased tooth.

### ANOTHER PAPER.

. . . Very many are cured by these medicines, but forty or fifty per cent. are only cured in one or two years.

There are two remedies required, one of holding the medicine in the mouth, and the other washing. The medicine which the dentists give the patients to hold in the mouth is this: They use pepper, gingers, juice, or the roots of keung (a tree whose flowers bloom and decay in a day) and decoct one of them into a decoction. They are of ferocious property, so that I do not know whether it can heal the sickness or not, but I know that when this medicine is held in the mouth, only a very little while, the mouth will swell up, and moreover patients feel more uneasy. The remedy of washing is the most wonderful of all. How do they wash the patients? They go to the hills to gather the buds of the mulberry plants, and put them in a mortar and pound them very fine, then they strain them through a cloth in order to take away the drugs. Now they put the juice in a basin and then begin to wash the patient's *eyes* with a piece of silk batting. When the juice is filtered they then change for the pure juice again, in the basin, to wash the patient's eyes, and do this twenty or more times. When I saw this I asked: "What do you do that for?" They answered that the germs of the teeth were drawn up to the eyes by the medicine and washed off. Accordingly I made an examination of the juice. Oh! I saw that there were germs moving in the juice which were large as hairs and of a very white color. They washed till I could find no germs in it, then stopped. Then the toothache was caused by the germs. Accordingly if the germs were washed off there should not be any more toothache. Why are the patients only cured in one or two years? Because, no doubt, the germs which they produced are not true germs, I think, but only to cheat for money.

[After mentioning other treatment (and that of using arsenic—Lu-sauk-tang) he says:] The receipt of Lu-sauk-tang is as follows: Burn the bones of the dog or man into ashes, and then mix them with some other stingy medicines and pound them very fine like powder. I am very sorry to say I cannot remember this receipt all right, for I cannot find my receipt.

ANOTHER.

"Teeth are the most important objects in human life." "If any animal has no teeth it will be very difficult to make him a living." "Teeth are very necessary to digestion, and are very important in another way; they aid to give a fine complexion to the personal appearance of anyone."—*Jour. of Brit. Dent. Association.*

